# Nanofiber Ion-Exchange Membranes for the Rapid Uptake and Recovery of Heavy Metals from Water

**DOI:** 10.3390/membranes6040059

**Published:** 2016-12-20

**Authors:** Nithinart Chitpong, Scott M. Husson

**Affiliations:** Department of Chemical and Biomolecular Engineering and Center for Advanced Engineering Fibers and Films, Clemson University, 127 Earle Hall, Clemson, SC 29634, USA; nchitpo@g.clemson.edu

**Keywords:** cadmium, electrospinning, membrane adsorber, microfiltration, water purification

## Abstract

An evaluation of the performance of polyelectrolyte-modified nanofiber membranes was undertaken to determine their efficacy in the rapid uptake and recovery of heavy metals from impaired waters. The membranes were prepared by grafting poly(acrylic acid) (PAA) and poly(itaconic acid) (PIA) to cellulose nanofiber mats. Performance measurements quantified the dynamic ion-exchange capacity for cadmium (Cd), productivity, and recovery of Cd(II) from the membranes by regeneration. The dynamic binding capacities of Cd(II) on both types of nanofiber membrane were independent of the linear flow velocity, with a residence time of as low as 2 s. Analysis of breakthrough curves indicated that the mass flow rate increased rapidly at constant applied pressure after membranes approached equilibrium load capacity for Cd(II), apparently due to a collapse of the polymer chains on the membrane surface, leading to an increased porosity. This mechanism is supported by hydrodynamic radius (R_h_) measurements for PAA and PIA obtained from dynamic light scattering, which show that R_h_ values decrease upon Cd(II) binding. Volumetric productivity was high for the nanofiber membranes, and reached 0.55 mg Cd/g/min. The use of ethylenediaminetetraacetic acid as regeneration reagent was effective in fully recovering Cd(II) from the membranes. Ion-exchange capacities were constant over five cycles of binding-regeneration.

## 1. Introduction

Although all living organisms require trace amounts of heavy metals such as cobalt, copper, iron, and manganese, non-essential heavy metals released in large quantities such as cadmium, chromium, mercury, lead, arsenic, and antimony are of great environmental concern [1]. Exposure to such metals can cause such serious health effects as reduced growth and development, cancer, organ damage, nervous system damage, and in extreme cases, death [2]. Many heavy metal pollutants are present in wastewaters that are discharged from metallurgical/metal manufacturing, electroplating and mining operations, printing, dye and paint, pulp and paper, textiles, and petrochemical operations, and from the manufacture of batteries and chemicals [3,4]. The growth of these industries results in direct or indirect discharge of larger amounts of heavy metals into the environment where they can accumulate in lethal quantities, particularly in developing countries that lack proper waste management protocols [3,4,5].

Substantial efforts have been undertaken to develop strategies to mitigate the amount of metal released to the environment by waste disposal and wastewater discharge. Many conventional wastewater treatment processes such as chemical precipitation, adsorption and ion exchange, and electrochemical deposition have been used to remove and recover heavy metals from impaired waters [2]. Additionally, newer approaches including membrane separation and electrodialysis have been developed to increase the level of metals removed from wastewaters [2,4].

Ion-exchange processes have been used widely to separate and recover specific metal ion impurities from wastewaters via the use of packed beds of ion-exchange resins. The most common cation exchangers in use are strongly acidic resin beads with sulfonic acid groups and weakly acidic resin beads with carboxylic acid groups. Separation occurs through the exchange of hydrogen ions (at pH < pKa) or cations such as sodium ions (at pH > pKa) on the resin with the metal cations in solution. However, the ion exchange resin process is limited in part by pressure drop across the resin bed during flow, which can be exacerbated by media deformation and non-uniform packing [6,7]. In several studies undertaken to correlate the pressure drop across packed beds of ion-exchange resin with flow rate, it was determined that the pressure drop must be known to design an ion exchange process effectively [8,9,10]. It was further determined that backwashing was required to minimize the pressure drop caused by resin fines and suspended solids [10].

The ion exchange resin process is also limited by mass transfer of ions to binding sites. Although it is possible to decrease the characteristic time for diffusion through the use of smaller porous beads, the pressure drop will increase accordingly [11]. To understand diffusion limitations in packed-bed resin processes, studies have been undertaken to elucidate the effect of flow rate (i.e., residence time) on the ion-exchange capacity. In a study on the use of a Purolite C100-MB cation exchange resin bed to remove copper(II) from aqueous solution, Hamdaoui [12] found that the removal depends upon the flow rate of the system. He also determined that the flow rate dependence is characterized by the need for a long residence time to achieve a high enough ion exchange capacity by providing more time for interaction between the metal and the resin [12]. In a similar experiment, Amberlite IRC-718 was used to elucidate the binding capacity of the resin for zinc(II). A higher capacity was measured at the lower flow rate, but reducing the flow rate also reduced productivity [13].

Membrane columns are an alternative to packed resin columns. A common configuration is to pack the column with a short stack of porous membranes with a large diameter to avoid high pressure drops [11]. Feed solution passes through the membrane bed, and since binding ligands are attached to the pore surfaces of the porous membrane, the diffusional path length of the target molecules (or ions in this case) to the functional groups is short [14]. Therefore, the transport of target solutes to the binding sites is controlled mainly by pressure-driven convective flow through the membrane [14,15]. As long as the residence time from convective flow is longer than the characteristic time for ion binding (i.e., Damköhler number, Da > 1), binding capacities are expected to be independent of flow rate. Membrane sheets can be cut to size and pre-packed into commercial filtration cells of various diameters. Scale up of membrane columns is linear and easy to do, unlike resin-packed beds [14,15].

Wada et al. studied copper chelation under flow conditions using a functional polymer grafted to porous polyethylene sheets as an alternative to conventional sorbents in bead form [14]. It was determined that porous sheets modified with iminodiacetate (dicarboxylate) chelating groups had a three-fold improved copper dynamic binding capacity at the same flow velocity than the DIAION^®^CR 11 chelating beads with the same functional groups [14]. Additionally, compared to conventional resin-based ion-exchange media, high-surface area ion-exchange membranes coated with polymer ligands exhibited a higher binding capacity and higher volumetric throughput [4,16]. For instance, it was reported that Amberlite IRC 748 ion exchange resins bind Cu(II) at flow rates of 10 bed volumes (BV)/h and a productivity of 0.13 mg Cu recovered/g resin/min (at an initial concentration of 317 mg/L), while functionalized nylon membranes could achieve flow rates up to 400 BV/h for Cu(II) and productivity of 2 mg Cu recovered/g membrane/min (at an initial concentration of 100 mg/L) [17,18].

This paper reports findings on the performance of nanofiber-based ion-exchange membranes for the rapid recovery of heavy metals from impaired waters. Membranes were prepared according to previous work [19]. The goals were to understand the role of flow rate on membrane dynamic binding capacity and productivity, and to develop a strategy to regenerate the membranes for metal ion recovery and membrane reuse. To attain these goals, we prepared macroporous cellulose nanofiber membranes by electrospinning and modified them by grafting poly(acrylic acid) (PAA) and poly(itaconic acid) (PIA) onto the nanofibers. The dynamic binding capacity of cadmium on the modified membranes was evaluated at different flow rates. Cadmium was selected for the study because it is a toxic metal of great toxicological concern, in that it is persistent and cannot be broken down into less toxic substances in the environment [20,21]. This metal is of such a concern that most countries have their own pollution control department to limit cadmium in all industrial effluents prior to disposal [21]. The role of the polymer coating type on both the dynamic binding capacities and the ion-exchange kinetics was analyzed, as was the polymer swelling behavior in the presence and absence of cadmium.

## 2. Experimental Section

### 2.1. Materials

The following chemicals and solvents were purchased from Sigma-Aldrich (St. Louis, MO, USA) and used as received: Alumina (an inhibitor remover), azobisisobutyronitrile (AIBN, 98 wt %), cadmium nitrate tetrahydrate ((Cd(NO_3_)_2_·4H_2_O), ≥99 wt %), cellulose acetate (CA, average Mn = 30,000 Da by GPC), diethyl ether (≥99%), ethylenediaminetetraacetic acid (EDTA, 99.4 wt %, powder), glycidyl methacrylate (GMA, 97 wt %), itaconic acid (IA, ≥99 wt %), poly(acrylic acid) (PAA, average Mw = 250,000 Da, 35 wt % in water). The following chemicals and solvents were purchased from Fisher Scientific (Waltham, MA, USA) and used as received: acetone (histological grade), 2-butanol (2-BuOH, 99%), dioxane (≥99%), nitric acid (90%, aq). Dimethylacetamide (DMAc, 99 wt %) and sodium hydroxide (NaOH, pellets, 97 wt %) were purchased from Alfa Aesar (Ward Hill, MA, USA). Chloroform (HPLC grade) was purchased from Honeywell (Morristown, NJ, USA).

### 2.2. Methods

#### 2.2.1. Preparation of Poly(glycidyl Methacrylate) (PGMA)

PGMA was prepared by radical polymerization of GMA using AIBN as initiator. GMA was dehibited by passing it through a column packed with alumina. The polymerization was conducted by adding dehibited GMA (10 g) and AIBN (1 g) in acetone (15 mL) at 40 °C under a nitrogen atmosphere and stirring with a magnetic bar. PGMA (Mw = 89,500 Da by gel permeation chromatography) obtained after a 2 h polymerization was purified by multiple precipitations (4 times) from acetone by the addition of diethyl ether. The PGMA was dried in a vacuum oven under 50 kPa overnight at room temperature.

#### 2.2.2. Preparation of Regenerated Cellulose Nanofiber Membrane Support

Regenerated cellulose nanofiber membranes were prepared according to previously established procedures [19]. Concisely, cellulose acetate in DMAc/acetone solvent was electrospun for 4 h using a voltage of 12.5 kV and a polymer solution flow rate of 0.3 mL/h. The relative humidity of the system was maintained at 65% to avoid bead formation on the fibers. The membranes were sintered thermally under an applied pressure and converted to regenerated cellulose nanofiber membranes by treatment with dilute NaOH solution. PGMA was coated on the regenerated cellulose nanofiber membrane from a solution in chloroform, and annealed under a vacuum. PGMA-coated regenerated cellulose nanofiber membranes (RC-PGMA) had an average thickness of 72 ± 13 µm.

#### 2.2.3. Preparation of Nanofiber Ion-Exchange Membranes

Again using a previously established procedure [19], PAA was precipitated by mixing the PAA solution with acetone. The mixture was centrifuged and the clear PAA gel was separated from the solution by decanting and was dried completely.

PIA was prepared by the radical polymerization of IA using AIBN as an initiator. Specifically, IA (10 g) in dioxane (40 mL) was added into an Erlenmeyer flask (150 mL). The quantity of the initiator AIBN was 1.6% by weight (0.16 g) in relation to the monomer. It was added to the solution containing IA in dioxane. The mixture was heated for 48 h at 60 °C, and acetone was added to the mixture in a separatory funnel to precipitate PIA and was left overnight in the hood. The product PIA (Mw = 7000 Da by intrinsic viscosity measurements) was separated from the monomer by vacuum filtration, washed three times with 50 mL of acetone, and dried overnight at 50 °C and 50 kPa in a vacuum oven.

PAA and PIA were dissolved in deionized water (DI water) to prepare 7 wt % polymer solutions, which were then sonicated for either 1 h or until they formed a homogenous solution. Each polymer solution was passed through a cellulose acetate sterile syringe filter (with a pore size 0.45 µm) purchased from VWR International (Radnor, PA, USA) to remove large size aggregates before use. Each RC-PGMA membrane was submerged in 4 mL of the filtered polymer solution for 5 min, removed from the solution, and annealed at 70 °C for 2 h. Thereafter, the membranes were washed three times (5 min per wash step) via immersion in 30 mL of water to remove any non-grafted polymer.

### 2.3. Membrane Morphology

The uniformity and quality of the fibers were examined by scanning electron microscopy (SEM model: S4800, Hitachi High Technologies America, Inc., Schaumburg, IL, USA). Representative 0.5 cm^2^ samples of the membranes were attached with carbon tape to aluminum stabs prior to the SEM measurements. The SEM measurements were performed at an accelerating voltage of 5.0 kV, current of 2 mA, and magnifications of 3000–12,000×. Average fiber diameters and fiber diameter distributions were determined from SEM images. Moreover, changes in the morphology of fibers due to sintering, hydrolysis, and modification of polymers were monitored by analysis of SEM images.

### 2.4. Performance Properties of Nanofiber Membranes

#### 2.4.1. Dynamic Binding Capacity and Regeneration

The PAA- and PIA-modified regenerated cellulose nanofiber membranes (RC-PGMA-PAA and RC-PGMA-PIA) were neutralized via immersion in a sodium hydroxide solution (0.05 M) for 1 h, cut into 45 mm diameter sections using a circular die, weighed, and placed into a 300 mL ultrafiltration stirred cell (Sterlitech Corporation, HP 4750 Stirred Cell, Kent, WA, USA). A cadmium solution with a concentration of 10 mg/L in DI water was prepared from Cd(NO_3_)_2_·4H_2_O and added to the ultrafiltration cell connected to an air cylinder. The permeate samples were collected every 5 min until fully loaded with Cd using feed pressures of 20.7, 34.5, and 48.3 kPa. Each permeate sample was weighed to determine mass flow rate, and Cd concentrations were measured to prepare breakthrough curves for evaluating the dynamic binding capacities. A 0.5 M aqueous solution of EDTA was prepared and pH adjusted to 6 for use as the regeneration reagent. The EDTA solution was passed through the membrane in the ultrafiltration cell and permeate samples were collected, weighed, and analyzed for Cd concentrations to prepare the regeneration curve. After regeneration to recover the Cd, sodium hydroxide solution (0.05 M, 10 mL) was passed through the membrane to ensure that the membrane was neutralized to the sodium carboxylate form. Finally, the membrane was rinsed by passing 20 mL of DI water through it before testing the next cycle. Five cycles of Cd binding and regeneration by EDTA were done at an applied pressure of 20.7 kPa. A single cycle was performed for each of the two other applied pressures.

Inductively coupled plasma optical emission spectroscopy (ICP-OES, Optima 5300 DV, Perkin Elmer, Waltham, MA, USA) was used to measure Cd(II) concentrations in permeate samples, with the peak area for each ion measured five times for each sample and calibration solution.

#### 2.4.2. Static Cadmium Binding Kinetic Study

Batch ion-exchange measurements were used to evaluate cadmium binding kinetics and to determine the static (i.e., equilibrium) binding capacities. Cadmium solutions (3 mL) with an initial concentration of 20 mg/L were placed in 20 mL vials. Next, PAA- and PIA-modified cellulose nanofiber membranes were cut into 10 mg pieces and placed into each vial. Experiments were conducted for contact times from 1 to 24 h in a shaker bath (22 °C, 100 rpm). ICP-OES was used to measure Cd(II) concentrations.

#### 2.4.3. Polymer Characterization in Solution

Dynamic light scattering (DLS, Model: Dawn Heleos-II, Wyatt Technology, Santa Barbara, CA, USA) was used to determine the hydrodynamic radius (R_h_) of PAA and PIA in DI water before and after Cd(II) loading. Cd(II) solutions with concentrations from 490 to 6250 mg/L in DI water (20 mL) were prepared and adjusted to pH 7 by NaOH solution. PAA (0.15 g) was dissolved in each Cd(II) sample and pH was again adjusted to pH 7 by NaOH. Separately, PAA and PIA (0.15 g) were dissolved in DI water (20 mL) and neutralized by NaOH to prepare controls. All solutions were analyzed to determine R_h_ of the polymer under different conditions. The light scattering analyses were performed every 3 s over the course of 5 min for each sample, to determine values with uncertainties representing one standard deviation.

## 3. Results and Discussion

### 3.1. Membrane Morphology

**Figure 1:**
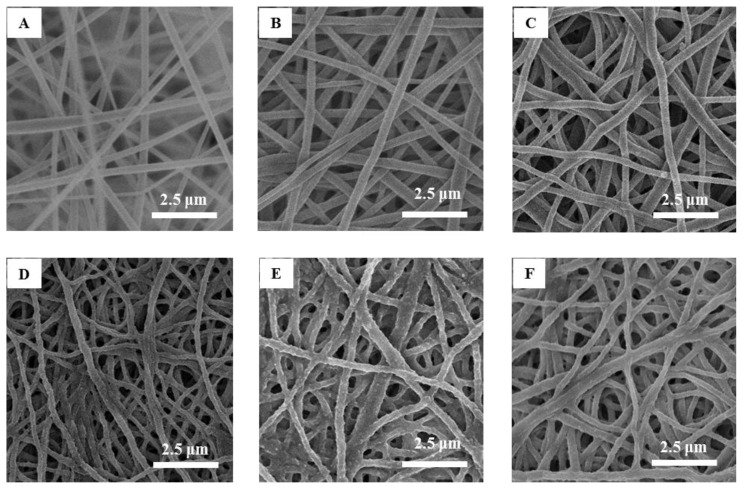
SEM images of cellulose acetate nanofiber mats before (**A**) and after (**B**) sintering; after hydrolysis (**C**); after modification by poly(glycidyl methacrylate) (**D**); and after modification by poly(acrylic acid) (**E**) and poly(itaconic acid) (**F**).

**Figure 2:**
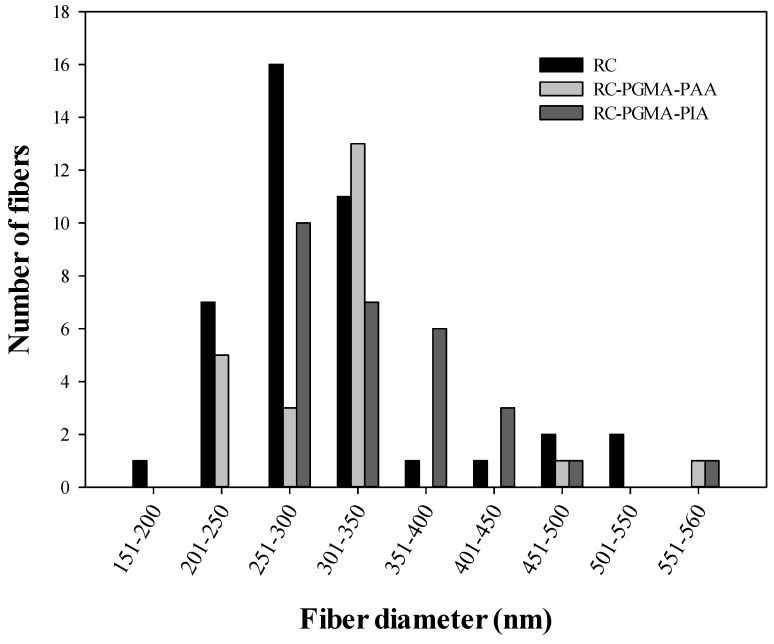
Nanofiber diameter distribution before and after modification by poly(acrylic acid) and poly(itaconic acid).

Figure 1 presents SEM images of cellulose acetate nanofiber membranes before and after sintering and hydrolysis (Figure 1A–C), after modification with PGMA (Figure 1D), and after coating with PAA (Figure 1E) and PIA (Figure 1F). The PAA and PIA thin films coat the surfaces of individual nanofibers and partially fill the pore spaces among nanofibers, causing an observed decrease in porosity [19]. It was found that the regenerated cellulose electrospun fibers before modification have an average diameter of 295 ± 84 nm. After modification with PAA and PIA, average fiber diameters were 299 ± 80 and 313 ± 79 nm, respectively. The fiber diameter distributions shown in Figure 2 demonstrate that even though fiber diameters tended to increase with polymer coating, average diameters are not different within uncertainties at 68% confidence level after being coated by PAA and PIA.

### 3.2. Membrane Performance Properties

#### 3.2.1. Dynamic Sorption-Regeneration Cycles for Cd(II) Ion Exchange on PAA- and PIA-Modified Nanofiber Membranes

The electrospinning technique was selected to produce membranes with a high surface area [22,23,24,25]. Polymeric ligands with carboxylate groups have been shown to be effective at complexation with trace heavy metals, most particularly cadmium in industrial wastewaters. In our previous study [19], PAA-modified regenerated cellulose nanofibers were used to successfully bind cadmium at equilibrium conditions.

**Figure 3:**
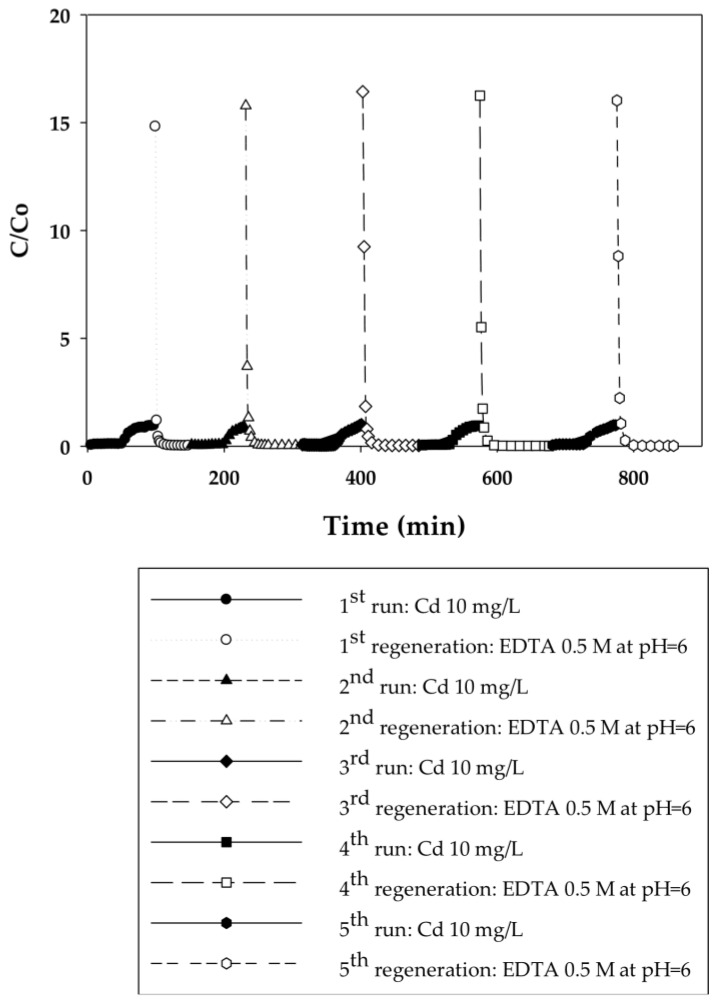
Breakthrough curves for Cd(II) ion-exchange on PAA-modified electrospun regenerated cellulose nanofiber membranes for five cycles at a constant applied pressure of 20.7 kPa. Regeneration was done with 0.5 M EDTA at pH 6.

**Figure 4:**
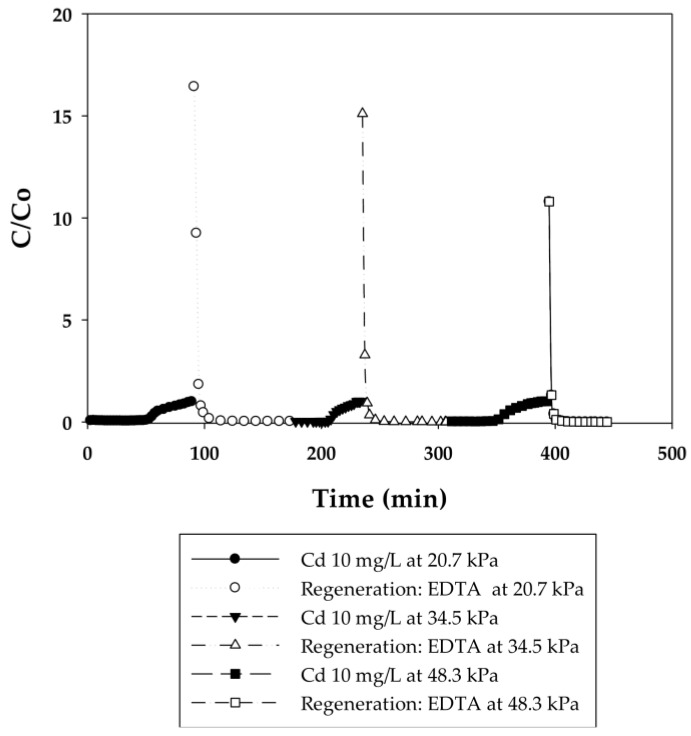
Breakthrough curves for Cd(II) ion-exchange on PAA-modified electrospun regenerated cellulose nanofiber membranes for three cycles at applied pressures of 20.7, 34.5, and 48.3 kPa. Regeneration was done with 0.5 M EDTA at pH 6.

**Figure 5:**
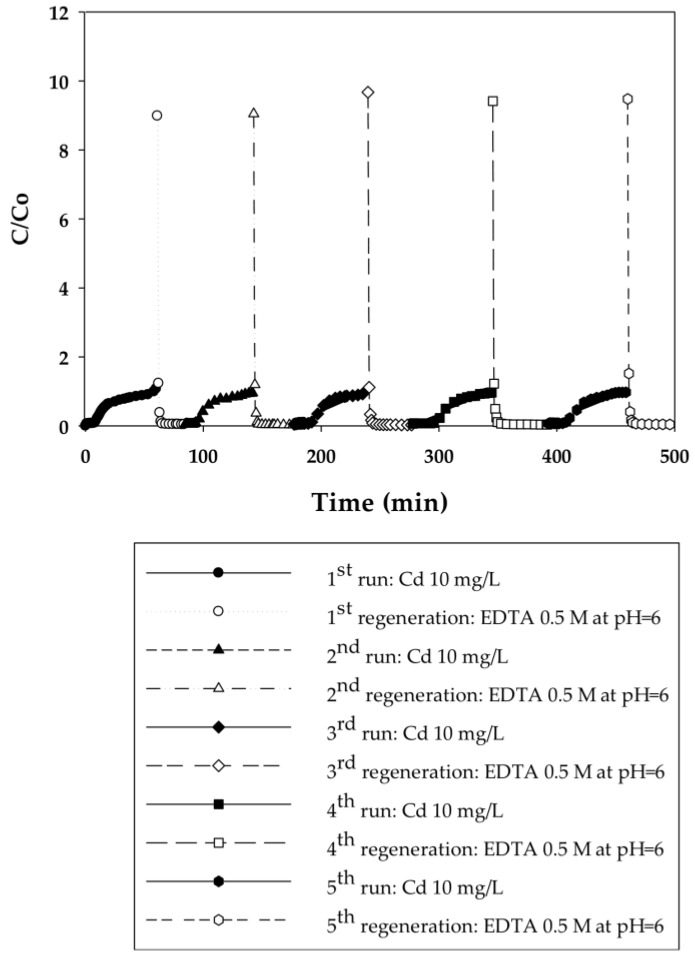
Breakthrough curves for Cd(II) ion-exchange on PIA-modified electrospun regenerated cellulose nanofiber membranes for five cycles at a constant applied pressure of 20.7 kPa. Regeneration was done with 0.5 M EDTA at pH 6.

**Figure 6:**
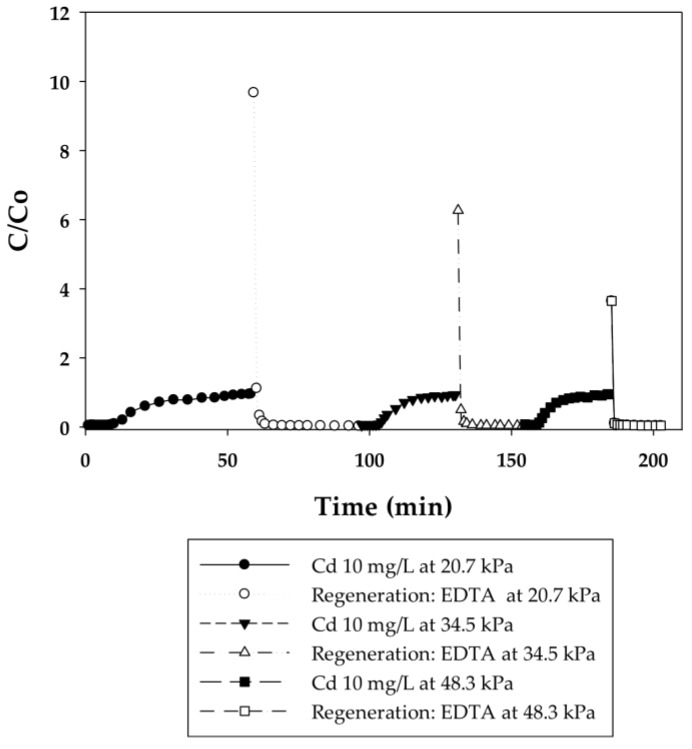
Breakthrough curves for Cd(II) ion-exchange on PIA-modified electrospun regenerated cellulose nanofiber membranes for three cycles at applied pressures of 20.7, 34.5, and 48.3 kPa. Regeneration was done with 0.5 M EDTA at pH 6.

In the current work, breakthrough curves were used to evaluate the dynamic binding capacities of PAA- and PIA-modified nanofiber membranes. A 10 mg/L solution of cadmium in DI water was used for measurements, as this is the maximum concentration in industrial wastewaters from electroplating processes [26]. To elucidate the effect of flow rate on dynamic binding, a low pressure of 20.7 kPa was initially applied to the system for five cycles and then increased to 34.5 and 48.3 kPa for two additional cycles. During the binding steps, experiments were stopped when a constant Cd(II) concentration of 10 mg/L was measured in the effluent. This condition indicates that membranes reached equilibrium with Cd(II) at that concentration, preventing any further uptake of Cd(II). Figure 3, Figure 4, Figure 5 and Figure 6 present breakthrough curves for all membranes and applied pressures. Dynamic binding capacity values were obtained by measuring the area to the left of these breakthrough curves at the point of 10% breakthrough (i.e., C/C_o_ = 0.10). This definition of dynamic capacity is standard, and consistent with the point at which ion loading would be stopped during operation [27]. Static binding capacities at 10 mg/L, defined as the maximum binding capacity at that concentration, were also measured from the entire area under the breakthrough curves.

In that EDTA, which is a strong chelating agent, is an efficient regeneration agent to elute Cd(II) from sorbents, 0.5 M of EDTA was used to regenerate membranes prior to reuse [28,29,30]. Although acids such as HNO_3_, H_2_SO_4_, or HCl could be used for regeneration in cases where the permeate will be returned back to the manufacturing process tank, EDTA is used commonly [31]. Furthermore, EDTA and metals in solution can be recovered by using methods discussed in the literature [32]. Permeate samples during regeneration were collected every 5 min to plot elution curves, also shown in Figure 3, Figure 4, Figure 5 and Figure 6. Here it is evident that EDTA is a good regeneration agent in that the Cd(II) permeate concentrations were initially quite high and rapidly declined until reaching zero. For an efficient regeneration agent, the high initial Cd(II) concentration is favorable because it indicates concentration of the metal ion as it is eluted from the membrane bed. After confirming that regeneration fully recovered Cd(II), the dynamic binding-regeneration process was undertaken for five duplicate runs at constant applied pressure of 20.7 kPa to demonstrate the reusability of the membranes. As indicated by the data in Table 1 and Table 2, the process was a success in that the binding capacities for both membrane types remained constant over the course of five cycles.

As also indicated in Table 1 and Table 2, the Cd(II) static binding capacity on the PIA-functionalized membrane was higher than the PAA-functionalized membrane. However, the dynamic binding capacity on the PIA-functionalized membrane was only 30% of its static binding capacity, which was lower than dynamic capacity on PAA membranes that corresponded to 40% of its static binding capacity. As expected, dynamic binding capacities were lower than static binding capacities, the causes of which were chromatodiffusion along the membrane bed in the direction of flow, a broad pore-size distribution, and load-dependent binding kinetics [27].

#### 3.2.2. Effect of Flow Rate on the Binding Capacity of Cd(II) on PAA- and PIA-Modified Nanofiber Membranes

Different applied pressures were used to study the effect of flow rate (i.e., residence time) on dynamic binding capacity. Figure 4 and Figure 6 show the breakthrough curves with the initial flow rates of 0.3, 0.45, and 0.6 mL/min corresponding to 140, 210, and 280 BV/h for the PAA membranes and 2, 3, and 4 mL/min corresponding to 950, 1420, and 1890 BV/h for the PIA membranes. Unlike conventional resin packed columns, the Cd dynamic binding capacities on both the PAA- and PIA-modified membranes shown in Table 3 and Table 4 persist after the increases in flow rate. This result is consistent with many studies that have shown that dynamic binding capacities often are independent of flow rate using membrane adsorbers [33,34], because sorption rates are not diffusion limited in macroporous membranes. The opposite is true for packed resin beds, where the diffusion time affects the dynamic binding capacities. These characteristics subsequently make the scale-up of the membrane process much easier.

A comparison of the membrane system flow rates (in bed volume per hour (BV/h) and volumetric flow rate per mass sorbent) and productivities (at 48.3 kPa) with those of commercial ion exchange resins in Table 5 shows the advantage of the membrane platform. The membrane columns can process feed with much higher volumetric flow rates due to the open pore structure of the membrane support. Productivity, representing the mass of Cd(II) bound per volume of sorbent per unit time, is another metric for comparing the experimental results with commercial products. Productivity is calculated using Equation (1):

(1) $$\mathrm{Productivity}=\frac{{\mathrm{DBC}}_{10\%}}{t_{\mathrm{break}}},$$

DBC_10%_ is the dynamic binding capacity and t_break_ is the time it takes to reach 10% breakthrough during the loading step. Our functionalized membranes performed better than ion exchange resins in terms of process flow rates and productivity. Factors limiting higher flow rates for the ion-exchange resins include high pressure drop and loss of dynamic capacity due to diffusional limitations. There is a limit to the productivity that can be achieved by the membrane platform. At high enough flow rates, the residence time for convective flow will approach the characteristic time for ion exchange. To understand this limit, information was needed on ion-exchange kinetics.

#### 3.2.3. Reaction Rate Analysis

**Figure 7:**
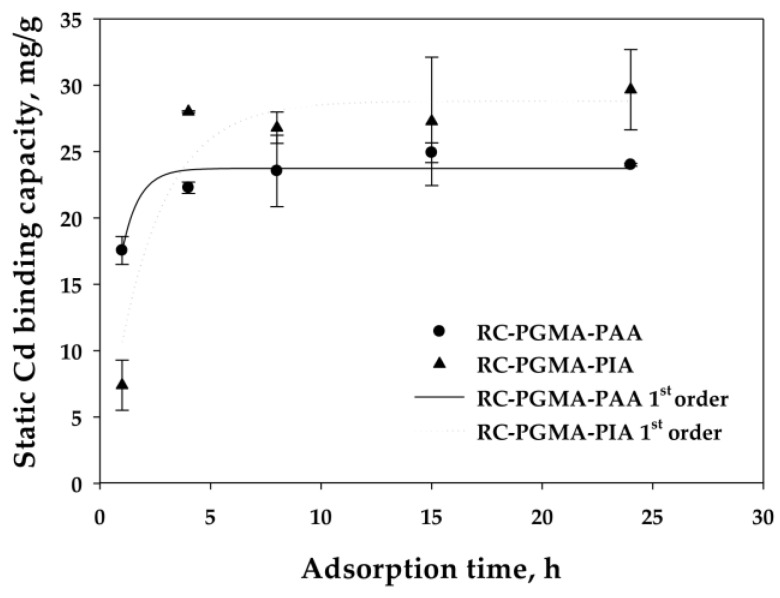
The ion exchange of the Cd(II) on PAA- and PIA-modified electrospun regenerated cellulose nanofiber membranes subjected to different contact times from 1 to 24 h. Symbols represent experimental data. Curves represent fits to the kinetic model. Error bars represent the standard deviation between two membrane samples at 68% confidence level.

It was shown that the dynamic binding capacities were independent of the flow rate, which indicates that the reaction rate was not limiting. However, the binding may become reaction rate limiting at high enough flow rates when the characteristic time is larger than the residence time for reaction. To determine the characteristic time for reaction, a study was conducted to measure the rate of reaction. Figure 7 presents the results of the kinetic binding experiments for the two membrane types. The kinetic binding studies were done at low initial concentrations of 20 mg/L to avoid the complication of load-dependent binding kinetics. This is the reason that the binding capacities in these experiments are lower than the maximum capacities shown in previous work, which evaluated uptake at higher equilibrium Cd concentrations [20]. A standard sorption rate model was applied to determine the kinetic binding constant for Cd binding on the membranes. Specifically, a pseudo-first-order reaction based on Cd ion, shown in Equation (2) and in integrated form in Equation (3) as established by Ho and McKay [39], was used to describe the binding kinetics [16,40,41,42].

(2) $$\frac{{\mathrm{dQ}}_{t}}{\mathrm{dt}}=k_{1}(Q_{e}-Q_{t})$$

(3) $$Q_{t}\;=Q_{e}(1-e^{-k_{1}t})$$

Q_t_ is the amount of sorbed metal per gram of the membrane at time t, Q_e_ is the equilibrium capacity (mg/g), and k_1_ (s^−1^) is the rate constant. It was found that the kinetic rate constant for Cd ion exchange on the PAA-modified membranes (3.7 × 10^−4^ s^−1^) was higher than that for the PIA-modified membranes (1.3 × 10^−4^ s^−1^) based on data regression from the kinetic model.

It is recognized that the efficiency of ion-exchange processes may be limited by binding kinetics. To assess whether the rate of ion exchange or the convective mass transport of the metal ion through the membrane bed was limiting the degree of metal uptake, the reaction Damköhler number (Da) was estimated using the following analysis. Da is a dimensionless quantity representing the ratio of the reaction rate to the rate of convective mass transport through a reactor and is defined by Equation (4) for a first-order reaction [43]:

(4) Da=kτ,

k is the reaction rate constant and τ is the residence time, or space time. A value of Da >1 indicates that the rate of ion exchange between the metal ion and functional group counterion is faster than the rate of convective transport of metal ion to the binding site. As Da decreases, less metal will bind due to insufficient space time for the ion exchange to occur (kinetically limited).

At the highest system flow rates studied for PAA- and PIA-modified membranes, the residence times were 13 s and 1.9 s. Based on the rate constants regressed from batch update data using the pseudo-first-order kinetic model, the calculated Da values from Equation (4) are 4.7 × 10^−3^ and 2.4 × 10^−4^ for PAA- and PIA-modified membranes, respectively. Such low values would suggest that the binding is kinetically limited; however, this finding is inconsistent with the observation that the dynamic binding is constant and non-zero over the range of flow rates studied. It thus appears that the rate constant values from the batch kinetic study may represent effective rate constants that depend on diffusion of ions to binding sites within the membrane pores, rather than the true intrinsic kinetic constants. Assuming for the moment that the rate limiting step is diffusion of metal ions from the main flow stream to binding sites at the pore surfaces, the characteristic time for sorption of metal ions can be estimated as r_p_^2^/D, where r_p_ is the diffusion path length and D is the diffusion coefficient of hydrated Cd in water which is reported to be 7.19 × 10^−10^ m^2^/s. Further assuming the average pore diameter of the nanofiber membrane is 1 µm (which correlates to nanofiber diameters of 200–500 nm) [44], the maximum effective diffusion path length is roughly equal to 0.5 µm (i.e., the average effective pore radius of the membranes). During loading, metal ions that must travel the full 0.5 µm to reach unoccupied binding sites will have a characteristic diffusion time of 3.5 × 10^−4^ s. Since this characteristic time is shorter than the lowest residence times for convective transport through the membrane, this scenario would suggest that convective flow is the rate-limiting mechanism. The assumption of rapid intrinsic reaction kinetics is therefore consistent with the observation that dynamic capacities are independent of flow rate over the range studied. Measurements of the true kinetic constants using higher flow rates through the short-bed membrane “reactor” would be needed to determine conditions where the binding becomes kinetically limited. However, it is possible that membranes will be damaged if the flow rate is too high.

#### 3.2.4. Polymer Behavior before and after Cd Ion Binding

**Figure 8:**
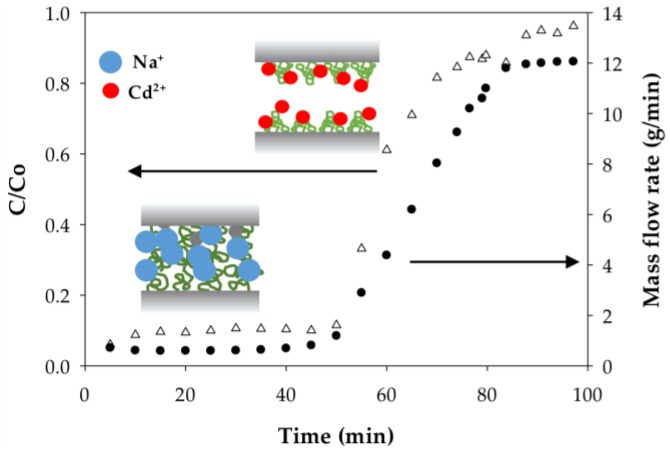
Dependence of the mass flow rate on degree of Cd(II) loading on PAA-modified electrospun regenerated cellulose nanofiber membranes. Inset figures illustrate differences in PAA chains swelling in the form of monovalent sodium ions and divalent cadmium ions.

**Figure 9:**
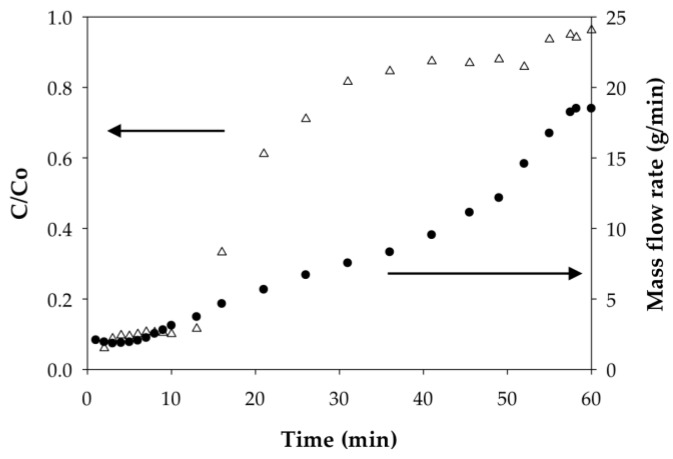
Dependence of the mass flow rate on the degree of Cd(II) loading on PIA-modified electrospun regenerated cellulose nanofiber membranes.

The weighing of permeate samples used to determine mass flow rates during dynamic analysis also uncovered something unusual; namely, the permeate mass flow rates at constant applied pressure for both the PAA- and PIA-modified membranes increased during each run as the membranes were loaded with a sufficient amount of Cd(II). Figure 8 and Figure 9 illustrate how the mass flow rate increased in relation to the Cd permeate concentrations (C/Co) for a single experimental run with PAA- and PIA- modified membranes. From a practical standpoint, a consequence of this finding is that accurate calculation of the static and dynamic binding capacities from the breakthrough curves requires using mass collections for permeate samples to account for the transient mass flow rates.

To understand the cause of the mass flow rate increase, we hypothesized that that polymer chains grafted to the membrane pore surfaces collapse (deswell) as a result of physical crosslinking between carboxylate groups due to ion exchange of monovalent Na ions with divalent Cd ions. Collapse of the chains leads to an increase in porosity and, thus, a higher flow rate. Incidentally, responsive polymer coatings have been used purposefully to create membranes with flow rates that can be adjusted using an external stimulus [45]. To test this hypothesis, polymer chain characteristics before and after binding Cd(II) were evaluated; specifically, we used dynamic light scattering to measure the hydrodynamic radius (R_h_) of PAA and PIA in solutions spiked with various amounts of Cd. As clearly indicated in the results in Table 6, the R_h_ of PAA decreased significantly with the addition of Cd(II) at an amount equivalent to 7.5% of total available ion exchange sites. R_h_ values further decreased slightly with an increase in the amount of Cd(II) until the polymer precipitated at high loading of Cd(II). Unlike PAA, only a slight decrease in R_h_ was measured for PIA with the addition of Cd(II) at low levels; however, like PAA, PIA was precipitated with the introduction of larger amounts of Cd(II). The precipitation of PAA and PIA at high Cd loads prevented measuring the R_h_ in these cases.

The findings shown in Table 6 that polymer R_h_ in solution decreases with the addition of Cd(II), eventually leading to precipitation, supports our hypothesis that Cd(II) binding causes a collapse of the grafted polymer chains on the membrane surfaces. Consequently, the pores open as illustrated in Figure 8, which, under conditions of constant applied pressure, yielded a higher mass flow rate. While the cause of the chain collapse is not known, it may be caused by physical inter- or intrachain crosslinks as the divalent ions of the Cd(II) exchange with two sodium ions on the grafted polymer ligands. Such crosslinks do not occur in the monovalent Na ion form. Close inspection of the PIA results in Table 6 shows that R_h_ in solution begins to increase slightly with an increase in the Cd(II) loaded just prior to precipitation. This finding suggests the formation of polymer aggregates due to crosslinks between polymer chains. The additional loading of Cd(II) caused a higher degree of crosslinking and large, insoluble aggregates that precipitated. In a study of poly(methacrylic acid) brushes placed in contact with aqueous solutions of Na(I), and Cu(II) salts, Konradi and Ruhe observed similar results: polymer layer swelling in the Na(I) system and a total collapse in the Cu(II) system [46]. They also determined that the difference in the polymer behavior was related to the different geometries of the carboxylate complex. Here, the divalent ions formed chelating complexes in a bridging bidentate configuration where the central ion is coordinated to both oxygen atoms of the carboxylate ligand, thus causing shrinkage in polymer brushes [46]. Understanding the underlying mechanism for swelling and collapse of the polymer coatings may have practical use in controlling the binding and regeneration cycles of metal removal and recovery processes.

## 4. Conclusions

This paper details the development of cation-exchange membranes with high productivity and durability for capturing heavy metals from impaired waters. Cadmium removal productivities of poly(acrylic acid) and poly(itaconic acid) modified electrospun cellulose nanofiber membranes were found to be 6–15 times higher than commercial ion-exchange resins, and the membranes could be reused at least five times without a decline in performance. Moreover, experiments with the polyacid-modified membranes determined that ion exchange was not diffusion limited, as is evident by flow rate independent dynamic binding capacities. The type of grafted polymer was also found to affect the ion-exchange performance of the membrane. Although the membranes with dicarboxylic acid functional groups exhibited a higher Cd static binding capacity than those with the monocarboxylic acid functional groups, they exhibited a lower Cd dynamic binding capacity. The grafted polymer swelling and collapse behavior on membrane pore surfaces between monovalent and divalent ions binding causes the increase of mass flow rate in the dynamic system. This polymer behavior may have practical use in controlling the binding and regeneration cycles of metal removal and recovery processes.

## Figures and Tables

**Table 1:** Static and dynamic Cd(II) binding capacities on PAA-modified electrospun regenerated cellulose nanofiber membranes for five cycles at constant applied pressure.

Cycle	Pressure	Static Binding Capacity	Dynamic Binding Capacity
	(kPa)	q_max_ (mg/g)	q_max_ (mg/g)
1	20.7	25.5	10.0
2	20.7	24.5	9.4
3	20.7	24.6	9.6
4	20.7	26.2	9.7
5	20.7	26.0	9.5

**Table 2:** Static and dynamic Cd(II) binding capacities on PIA-modified electrospun regenerated cellulose nanofiber membranes for five cycles at constant applied pressure.

Cycle	Pressure	Static Binding Capacity	Dynamic Binding Capacity
	(kPa)	q_max_ (mg/g)	q_max_ (mg/g)
1	20.7	37.5	5.5
2	20.7	34.5	9.6
3	20.7	37.1	8.2
4	20.7	33.9	9.5
5	20.7	33.6	10.2

**Table 3:** Static and dynamic Cd(II) binding capacities on PAA-modified electrospun regenerated cellulose nanofiber membranes at different applied pressures.

Cycle	Pressure	Static Binding Capacity	Dynamic Binding Capacity
	(kPa)	q_max_ (mg/g)	q_max_ (mg/g)
1	20.7	24.6	9.6
2	34.5	25.9	10.6
3	48.3	25.7	9.7

**Table 4:** Static and dynamic Cd(II) binding capacities on PIA-modified electrospun regenerated cellulose nanofiber membranes at different applied pressures.

Cycle	Pressure	Static Binding Capacity	Dynamic Binding Capacity
	(kPa)	q_max_ (mg/g)	q_max_ (mg/g)
1	20.7	34.6	9.6
2	34.5	32.9	7.9
3	48.3	33.3	8.2

**Table 5:** Comparison of ion-exchange membranes and commercial ion-exchange resins.

Ion Exchange Materials	q_max_	Permeability	Flow Rate		Productivity (C_o_ = 10 mg/L)
	mg Cd/g	L/m^2^/h/bar	BV/h	mL/min/g	mg Cd/g/min
Cellulose/alginic acid IEX membrane [35]	44.4	8.0	-	-	-
Chitosan/CA IEX membrane [16]	43.8	7.7	-	-	-
Dowex 50W [36]	277	-	0.5	-	-
Duolite CT-73 [37]	106	-	10	-	-
Amberlite 200 [37]	225	-	40	-	-
Amberlite XAD-7/Cyanex-301 [38]	-	-	-	0.64	3.5 × 10^−2^
RC-PGMA-PAA [19]	163	400	280	12	2.2 × 10^−1^
RC-PGMA-PIA	222	500	1890	80	5.5 × 10^−1^

**Table 6:** Hydrodynamic radius measurements for PAA and PIA. Percentages indicate the percentage of carboxylate groups in Na form and Cd form at constant pH = 7.

% Polymer in Na and Cd forms	100% Na + 0% Cd		92.5% Na + 7.5% Cd		85% Na + 15% Cd		75% Na + 25% Cd		50% Na + 50% Cd		0% Na + 100% Cd	
	R_h_ (nm)	SD	R_h_ (nm)	SD	R_h_ (nm)	SD	R_h_ (nm)	SD	R_h_ (nm)	SD	R_h_ (nm)	SD
PAA	67	0.67	11	0.21	9.4	0.08	10	0.10	9.4	0.04	*	-
PIA	62	0.81	5.5	0.04	6.1	0.01	7.4	0.01	*	-	*	-

* Polymer precipitated under these conditions.
